# Role of microsatellites in genetic analysis of *Bombyx mori *silkworm: a review

**DOI:** 10.12688/f1000research.20052.1

**Published:** 2019-08-13

**Authors:** Julian David Trochez-Solarte, Ximena Ruiz-Erazo, Martha Almanza-Pinzon, Giselle Zambrano-Gonzalez

**Affiliations:** 1 Agropecuary Sciences Department, Production Integrated Systems Research Group (SISINPRO), Faculty of Agricultural Sciences, University of Cauca, Popayán, Cauca, 190017, Colombia; 2 Biology Department, Geology, Ecology and Conservation Research Group (GECO), Faculty of Natural Sciences and Education, University of Cauca, Popayán, Cauca, 190002, Colombia

**Keywords:** Bombyx mori, silkworm, molecular marker, sericulture, Simple Sequence Repeats, SSR.

## Abstract

In the genome of *Bombyx mori* Linnaeus (1758), the microsatellites, or simple sequence repeats (SSR), feature among their particular characteristics a high adenine and thymine (A/T) content, low number of repeats, low frequency, and a grouping in "families" with similar flanking regions. Such characteristics may be the result of a complex interaction between factors that limit the size and dispersion of SSR loci—such as their high association with transposons—and mean that microsatellites within this taxon suitable as molecular markers are relatively rare. The determination of genetic profiles in populations and cell lines has not been affected owing to the high level of polymorphism, nor has the analysis of diversity, structure and genetic relationships. However, the scarcity of suitable microsatellites has restricted their application in genetic mapping, limiting them to preliminary identification of gene location of genes or quantitative trait loci (QTLs) related to thermotolerance, resistance to viruses, pigmentation patterns, body development and the weight of the cocoon, the cortex, the pupa and the filament. The review confirms that, as markers, microsatellites are versatile and perform well. They could thus be useful both to advance research in emerging countries with few resources seeking to promote sericulture in their territories, and to advance in the genetic and molecular knowledge of characteristics of productive and biological interest, given the latest technological developments in terms of the sequencing, identification, isolation and genotyping of SSR loci.

## Introduction

Domesticated around 5,000 years ago, the silkworm, *Bombyx mori* L. is the basis of sericulture, an agroindustrial activity that involves the breeding of silkworms, cultivation of *Morus spp*. mulberry plants as the sole source of food, and industrial processing of cocoons to produce natural silk yarn ^1^. *B mori* is an ideal organism as an experimental animal for genetic and biological research. Silkworms are easily reared and produce a genetically uniform population. This, added to their economic importance, has made *B. mori* one of the most widely studied insects ^2, 3^. As a result, genetic materials of agronomic and scientific interest have been identified, characterized and conserved ^4^ using a variety of molecular markers, prominent among them microsatellites, or simple sequence repeats (SSRs).

Microsatellites are regions of DNA in which a sequence or motif between 1 and 6 bp is repeated in tandem 5 to 100 times, the number of repeats of the same locus being highly variable both inter- and intra-populationally. They are also ubiquitous, distributed uniformly in eukaryotic genomes ^5^. SSR loci are usually flanked by regions of unique sequence, which allows PCR amplification with specifically designed primers and determination of the specific genetic profile or genotype of an individual for several loci through the pattern of bands displayed on capillary sequencing equipment ^5^. These markers are codominant and highly polymorphic. They have greater reproducibility and band comparability, are less sensitive to contamination by foreign DNA (due to their specificity) and can be amplified using fragmented or partially degraded DNA, because of the reduced size of the loci ^6^.

These molecular markers have been a useful tool in sericulture, especially in the management, characterization and conservation of materials in germplasm banks ^7, 8^ and in the development of genetically improved materials ^9^. They have been particularly difficult to apply, however, to genetic mapping, mainly due to the low frequency of loci suitable as molecular markers in the *B. mori* genome. Their contribution in this area has therefore been restricted to preliminary mapping of some genes and QTLs related to characteristics of productive interest ^10^ or to processes of *B. mori* body development and pigmentation ^11, 12^.

The review focuses on the description and analysis of the specific characteristics of microsatellites in the *B. mori* genome, the role these repetitive DNA regions have played as molecular markers in this organism, and how these would play an important role in future in the genetic analysis, conservation and use of silkworm germplasm.

## Characteristics of silkworm genome microsatellites

Microsatellite characteristics—distances between loci, abundance, distribution, motif and the average number of repeats they comprise—may vary among taxa ^13^. The *B. mori* microsatellites feature average distances of between 49 and 161 kb ^14, 15^, their genome coverage is only 0.31% ^16^ and they have a cloning efficiency of between 0.77 and 3.5% ^14, 15^. Together, such values indicate that microsatellites in the *B. mori* genome are rare. The pattern is not unusual however, having been observed in other insect species, including several in the *Drosophila* genus ^17, 18^.

SSR loci with mononucleotide-like motifs represent 60% of the microsatellites of the *B. mori* genome, high compared to other insect species ^17^, and are formed by a low number of repeats, nine on average among the different repeat motifs. Loci of more than 15 repeats are scarce, except for mononucleotide motifs ^16, 17^. Harr and Schlötterer ^19^ report that *Drosophila melanogaster* Meigen (1830) also has short SSR loci on average (<15 repeats), because the greater the length, the greater the tendency of the *D. melanogaster* microsatellites to mutations that decrease the number of repeats instead of increasing it. This is speculated to be the result of interaction between factors related to DNA repair and replication mechanisms.

The *B. mori* genome has a comparatively high composition of regions rich in adenine and thymine (A/T). Moreover, the microsatellites that include a high proportion of these bases are the most abundant, constituting approximately 40% of the dinucleotide motifs and 28.3% of the total ^20^. Such a predominance has not been seen in other organisms ^16, 20^.

Transposons appear to play a key role in the evolution of microsatellites in the *B. mori* genome ^21^. Of the *B. mori* SSR loci, 35% are grouped into "families" with flanking regions similar in sequence. This is due to their association with transposons ^17^, mobile genetic elements capable of replicating themselves and associated regions. Transposons account for 35–43.6 % of the *B. mori* genome ^22, 23^. For Meglécz *et al.* ^17^, this high association suggests one of two scenarios: transposons might favor the development of microsatellites in their vicinity, or promote their formation at the moment of transposition. It is possible, however, that they have no part to play in the genesis. The SSR loci could develop independently, but have a structure that favors the insertion of mobile elements ^24^.

The characteristics of the *B. mori* microsatellites—low frequency, low average number of repeats, and grouping in families—are common to the Lepidoptera order. They explain the difficulties in isolating single copy microsatellites ^17, 18, 25, 26^, but the advance in high-throughput sequencing techniques and graph-based cluster analysis ^27^, as well as screening against transposons elements in the isolation procedure ^28^ may in future facilitate identifying suitable microsatellites for genetic studies in species in this taxon.

Zhang ^21^ suggested that the characteristics of Lepidoptera microsatellites imply that, in their respective genomes, most have experienced a recent development and multiplication, in the different lepidopteran species. This may mirror that reported in *D. melanogaster* ^19^, characterized because its long SSR loci are of recent origin and have short prevalence periods.

Microsatellite evolution is extremely complex ^24^. The characteristics in *B. mori* and the Lepidoptera, as well as in other organisms ^29^, appear to result from interaction in the genome between the mutations or events that cause them and the factors that obstruct or limit their development, with a balance in favor of the latter in silkworm. The key to the low average number of repeats and low frequency of SSR loci in the *B. mori* genome may be the abundance of A and T bases, and of microsatellites composed of these bases. Regions rich in A/T have a higher frequency of double-strand breaks in the DNA, which can induce and facilitate both the loss of nucleotides during non-homologous recombination ^30^ and the insertion of transposons able to divide an SSR locus in two and interrupt its development ^24, 31, 32^. This could explain why A/T-rich microsatellites in *B. mori* have a lower average number of repeats, as reported by Zhan *et al.* ^20^.

Additionally, the presence in *B. mori* of an efficient DNA mismatch repair mechanism, the system in charge of correcting the incorrect incorporation of bases ^29^, could counteract replication slippage, the major mutational mechanism in explaining the origin and evolution of repetitive DNA regions ^24^.

## Applications of microsatellites

### Construction of DNA profiles

Microsatellites have proved a useful tool for generating band patterns that enable discrimination of silkworm lines. Kim *et al.* ^8, 33, 34^ found that 25–28% of the amplified alleles are specific to the lines, due to which a small number of microsatellites, one to three, allow identifying approximately 20% of the analyzed materials without resorting to the genotyping of other loci. Hou *et al.* ^35^ and Chandrakanth *et al.* ^36^ likewise indicate that the analyzed genotypes are homozygous for a substantial part of the microsatellites used. Since each locus is multiallelic, those that amplify as a single band in each line are powerful tools for identification, as demonstrated by Li *et al.* ^37^ when discriminating between two closely related lines identical in their morphological characteristics: Dazao and P50.

DNA fingerprinting also represents a tool for the identification of insect cell lines. Between 3 and 8 microsatellites have therefore been used to generate profiles of cell lines susceptible to the nuclear polyhedrosis virus of *B. mori* (BmNPV), developed to study replication and expression mechanisms of the virus ^38– 40^. McIntosh *et al.* ^41^ obtained stable DNA profiles even after performing 200 subcultures by using coding regions (aldolase, prolactin receptor, interleukin-1β) as molecular markers. Microsatellites are less stable, however, due to their high mutation rate—between 10 ^-4^ and 10 ^-6^ per locus per generation ^42^. Thus, for future identification of cell lines, it is advisable to evaluate and select SSR loci that present relatively low mutation rates.

### Analysis of genetic diversity

Sericulture depends on the strategic use of silkworm germplasm to develop hybrids with high yields of silk that resist or tolerate disease and adverse climatic conditions ^43^, based on knowledge of the extent and distribution of the genetic diversity available in both the domesticated silkworm *B. mori* and its wild relative *Bombyx mandarina* Moore (1872). In this context, between 500 and 700 microsatellites were developed in *B. mori*, of which 5 to 27 markers have been used for analyzing genetic diversity ( Table 1).

**Table 1.** Studies of genetic diversity conducted in *B. mori* using microsatellites.

Reference	SSRs ^†^ and alleles	PIC ^‡^	Genotypes and groups	Results
Reddy *et al.*, 1999 ^14^	15 and 113	-	13 and 2	Identification of specific alleles of lines with diapause and without diapause.
Zhang *et al.*, 2005 ^49^	27 and 146	0.57-0.88	12	The diversity in highest in *B. mandarina* populations, followed by Chinese, Japanese and European lines of *B. mori*.
Li *et al.*, 2005 ^37^	26 and 188	0.12-0.89	31 and 7	Groupings with mixtures of genotypes of different types of voltinism.
Hou *et al.*, 2007 ^35^	35 and 467	0.30-0.92	96	Groups composed of mixtures of genotypes with different types of voltinism and geographic origin. The greatest diversity in lines of Chinese origin.
Qian *et al.*, 2007 ^51^	11 and 106	-	40	The groupings coincide with differences in geographical origin and voltinism.
Vijayan *et al.*, 2010 ^53^	7 and 49	0.10-0.40	13 and 2	Genotypes with contrasting characteristics could be crossed to obtain hybrid vigor in their offspring.
Kim *et al.*, 2010 ^33^	9 and 68	0.06-0.86	54	The groupings do not coincide with phenotypic characteristics of the lines. On detecting 17 unique alleles, it was possible to identify 14 lines.
Thiyagu and Kamble, 2011 ^52^	10 and 139	-	10 and 4	The grouping coincided with differences in geographical origin and silk productivity of the lines evaluated.
Kim *et al.*, 2012 ^8^	8 and 76	0.34-0.82	85	On detecting 22 unique alleles, it was possible to identify 19 lines. The groups formed do not coincide with known phenotypic characteristics.
Chu and Peng, 2013 ^54^	22	0.0-100	23 and 2	The lines were divided into 2 groups.
Chandrakanth *et al.*, 2014 ^36^	15 and 54	0.16-0.75	10 and 4	The groups coincide with the geographical origin, and the subdivision coincides with the color or shape of the cocoon.
Furdui *et al.*, 2014 ^7^	5 and 31	0.35-0.67	15	20% of the genetic variance is between genotypes. Lines with wide genetic distances and contrasting characteristics are useful for developing new hybrids.
Kim *et al.*, 2014 ^34^	8 and 73	0.37-0.77	78	The groupings do not coincide with the phenotypic characteristics of the lines. On detecting 19 unique alleles, it was possible to identifying 16 lines.

† SSRs: microsatellite loci used (Simple Sequence Repeats)

‡ PIC: polymorphic information content (PIC) ranking of microsatellites within the study

Miao *et al.* ^15^ and Zhan *et al.* ^20^ discovered that the genome of *B. mori* lines with contrasting characteristics is similar, in terms of the low percentage of polymorphic SSR loci, ranging between 17% and 24% compared, for example, with 85% for the European bee, *Apis mellifera* ^44^, and 55% in laboratory rats ^45^. Together, these data attribute the origin of *B. mori* to a single domestication event from a reduced population of *B. mandarina* ^15^. Xia *et al.* ^46^, however, on conducting a complete genome analysis on domesticated lines and wild individuals report that *B. mori* harbors 83% of the genetic variability of wild populations, indicating that the origin was possibly not limited to a reduced population or a single domestication event ^43^.

The low percentage of polymorphic SSR loci found among domesticated lines of *B. mori* should not be interpreted as evidence of the reduction of genetic diversity with respect to wild populations. The low figure could instead be the result of size homoplasy, a process of change by which convergent mutations cause microsatellites, belonging to different lineages, to have the same length in base pairs ^47^.

Size homoplasy is favored when mechanisms are present that neutralize elongation of the microsatellites and limit the number of repeats, since possible alleles are reduced and mutations are more likely to converge in the same length. These conditions appear to be present in the *B. mori* genome, in which most microsatellites are of reduced size ^16, 17, 20^. In 2016, De Barba *et al.* ^48^ proposed a new method for genotyping microsatellites using high-throughput sequencing; this would allow direct access to the microsatellite sequences in *B. mori* and evaluate whether the low percentage of polymorphic loci is due to the presence of size homoplasy.

Although *B. mori* has not experienced a drastic reduction in genetic diversity compared to *B. mandarina*, the wide genetic distances between domesticated and wild populations ^49^ indicate that these species have a marked genetic differentiation and distant relationships. This is likely due to the absence of genetic flow, a result of the inability of *B. mori* to fly and to survive without human intervention ^50^. Thus, *B. mandarina* represents a potential unique source of genetic material for sericulture ^43^.

Cluster analyzes to determine the relationships between *B. mori* lines provide contradictory results. Reddy *et al.* ^14^, Qian *et al.* ^51^, Thiyagu and Kamble ^52^ and Chandrakanth *et al.* ^36^ report that grouping the materials based on the microsatellites analyzed corresponded to type of voltinism, geographical origin, silk productivity, color or shape of the cocoon. However, the groups formed in studies that analyzed a larger sample of germplasm—69 lines on average, compared to 18 in the works cited above—exhibit mixtures of genotypes with variability in these characteristics ^8, 33– 35, 37^.

Several different scenarios may explain why *B. mori* genotypes, with apparently diverse traits, are grouped together, the main one being the hybridization that has historically been used, in pure lines, to perform the introgression of genes that increase silk yield or survival rate in various environmental conditions ^55– 58^. This would alter their phenotypic characteristics but, due to backcrossing, they would maintain a similar—or even the same—genotype, depending on the microsatellites analyzed.

Various pure lines may also share an origin in the same ancestral population of *B. mandarina*, but would have been selected to express different phenotypes, maintaining a high similarity at the genotypic level ^7, 37, 59^. This might also explain why pure lines with similar characteristics are not always grouped, given that they would have a distant relationship, but would have been selected to express similar traits ^35, 37^.

Traditional genetic improvement in *B. mori* implies the use of phenotypic characteristics or geographical origin to differentiate and select parents with contrasting characteristics with which to perform crosses. However, due to the scenarios already mentioned, these characteristics do not always make it possible to accurately determine genetic relations between materials ^60^. Microsatellites thus represent an important tool for making accurate estimates of genetic diversity and relationships in order to develop genetically improved materials (hybrids), bearing in mind that, comparatively, performance is better than that of other markers such as RAPDs, RFPLs and ISSRs ^6^.

Microsatellites not only represent a marker with a good performance in analysis of genetic diversity in *B. mori*. They constitute a versatile tool that can be adjusted to meet the research requirements and the resources available. For example, they can potentially be genotyped with high-throughput sequencing for greater accuracy ^48^ and even be identified and analyzed simultaneously with SNPs to strengthen inferences about diversity, structure and genetic relationships ^61^. However, they can also be identified by means of polyacrylamide gels or capillary sequencing equipment when fluorescently labeled ^5^. As such, they provide accessible and profitable information for managing germplasm banks and promoting regional initiatives in developing countries that do not have the resources to access cutting-edge technology, yet view sericulture as an opportunity to generate employment and improve the conditions of the rural population ^1^.

### Linkage maps

Determination of the relative positions of microsatellite markers in the chromosomes of the *B. mori* genome began with the low-density linkage map developed by Prasad *et al.* ^16^. The medium density one constructed by Miao *et al.* ^15^ followed, with an average distance between markers of 6.3 cM and 29 linkage groups. Zhan *et al.* ^20^ subsequently increased the density of this map using new lines and mapping populations of *B. mori*, decreasing the average distance between markers to 4.8 cM ( Table 2).

**Table 2.** Data summary of linkage maps with microsatellites in *B. mori*.

Reference	Number of LGs ^†^	Individuals analyzed	Number of markers		
			Total	SSRs ^‡^	Others
Prasad *et al.*, 2005 ^16^	8 [1] ^§^	60 BF1 ^¶^	29	29	0
Miao *et al.*, 2005 ^15^	29 [26]	189 BF1	547	518	29
Nagaraja *et al.*, 2005 ^62^	1	55 BC1M ^††^	16	2	14
Miao *et al.*, 2008 ^63^	1	188 BC1M	8	6	2
Zhan *et al.*, 2009 ^20^	30 [28]	188-190 BF1	692	692	0

^†^LGs: linkage groups

^‡^SSRs: microsatellite loci (Simple Sequence Repeats)

^§^Numbers in square brackets indicate linkage groups assigned to established linkage groups

^¶^BF1: backcross

^††^BC1M: backcross with F1 male

The density achieved with the linkage map of SSR markers is below the results expected by the authors due to the high homology (low percentage of polymorphic loci) between the loci of the *B. mori* lines used to generate the mapping populations ^15, 20^. Nevertheless, the resolution is sufficient to carry out the preliminary gene screening ( Table 3) and identification of QTLs.

**Table 3.** Genes mapped using microsatellites in *B. mori*.

Name	LG ^†^	Expression or function	Reference
*nsd-Z*	15	Resistance to the virus of densonucleosis	Li *et al.*, 2006 ^70^
*Sel*	24	Yellow cuticle	Miao *et al.*, 2007 ^71^
*Xan*	24	Yellow cuticle	Miao *et al.*, 2007 ^71^
*Bmabd-A*	6	Additional limb pair	Xiang *et al.*, 2008 ^72^
*C*	12	Yellow pigmentation in cocoons	Zhao *et al.*, 2008 ^73^
*I*	9	Prevents absorption of carotenoids	Li *et al.*, 2008 ^74^
*Dtf*	15	Resistance to fluorinated compounds	Bai *et al.*, 2008 ^75^
*Bm-iAANAT*	18	Larvae and pupae with dark pigmentation	Dai *et al.*, 2010 ^76^; Zhan *et al.*, 2010 ^77^
*nlw*	13	Scaleless wings	Wang *et al.*, 2010 ^78^
*KN*	8	Thermotolerance	Zhao *et al.*, 2010 ^79^
*Ng*	12	No glue eggs	Zhao *et al.*, 2011 ^80^
*BmAntp*	6	Identify thoracic segments	Chen *et al.*, 2013 ^81^
*E ^Cs^-l*	6	Control abdominal segment development	Chen *et al.*, 2013 ^82^
*BmEP80*	10	Egg dehydration	Chen *et al.*, ** 2013 ^83^
*+P*	2	Formation of larval marks	Wei *et al.*, 2013 ^84^
*BmADC*	11	Black pupae	Dai *et al.*, 2015 ^11^
*BmLanB1-w*	13	Cell adhesion in wing tissues	Tong *et al.*, 2015 ^85^
*BmCPG10*	5	Regulation of molt	Wu *et al.*, 2016 ^12^
*Bmscarface*	23	Regulation of body shape	Wang *et al.*, 2018 ^86^

†LG: linkage group where the gene is located

Exclusive linkage maps for the Z chromosome were developed by Nagaraja *et al.* ^62^ and Miao *et al.* ^63^ with the purpose of contributing to identification of genes related to control of the duration of larval stages, diapause, moltinism, body size and color, etc., and to analyze the role played by characteristics linked to sex in evolutionary processes in Lepidoptera, and differentiation of geographic races ^64^.

### Identification of genes and QTLs

Identification of SSR markers linked to genes was carried out in order to understand the molecular basis of characteristics of agronomic and scientific interest. The SSR loci identified by Miao *et al.* ^15^ and Zhan *et al.* ^20^ enabled the identification of genes related to characteristics such as thermotolerance, resistance to the Z strain of the virus of densonosis in *B. mori*, tolerance to fluorinated compounds, and absence of wing scales; studies that were used to develop improved lines with marker-assisted selection ^9, 65, 66^. Genes related to pigmentation patterns were also identified, in cocoons and larvae, and in development processes such as the formation of extremities, thoracic segments, cell adhesion and regulation of molting ( Table 3).

Microsatellites have also allowed tracking of QTLs in *B. mori* related to weight of cocoon, cortex, pupa and filament ^10, 20^, which are mainly located on chromosome 1 where they are strongly linked to SSR loci (LOD>11.0), contribute significantly to phenotypic variation (30 %) and have a simultaneous effect on the above characteristics. ** The location of the QTLs in chromosome 1 was delimited to a region of 290 kb, in which 12 candidate genes were identified that will allow study of the molecular mechanisms underlying these characters of agronomic interest in *B. mori* ^10^. Additionally, Gao *et al.* ^67^ discovered that Bombyx mori Nuclear Polyhedrosis Virus (BmNPV) resistance is a polygenic characteristic.

Linkage maps with microsatellites have allowed us to advance in the knowledge of characteristics of economic interest in *B. mori*, as well as in the Lepidoptera genetic architecture. However, Xu *et al.* ^68^ and Li *et al.* ^69^ indicate that the most used models have not been adjusted according to the particular genetic characteristics of the silkworm, because they ignore the effects of gender and the presence of aquiasmatic meiosis, a process that implies the absence of chromosomal cross-linking in the germinal line of the females. As a result, these authors have proposed models that allow correcting the potential biases and lack of precision of the most used methods for mapping QTLs in *B. mori*. Xu *et al.* ^68^ propose a statistical model to analyze QTLs in F2 populations, while Li *et al.* ^69^ focus their method on the use of backcrossing and the rational selection of mapping populations to first identify the chromosomes with QTLs and subsequently their position.

## Conclusions and future work

The microsatellites in *B. mori* have particular characteristics such as low frequency, low average number of locus repeats and grouping into "families". These are shared by Lepidoptera and seem to indicate that factors or mechanisms exist within the genome of this taxon that limit growth and stability of these repetitive DNA regions and their used as single copy loci molecular markers. Such as an abundance of loci SSR rich in A/T susceptible to double strand breaks and loss of repeats, as well as the possible existence of efficient DNA repair mechanisms that avoid the incorrect incorporation of bases (DNA mismatch repair) and the high association with transposons that would cause the formation of groups of loci with high similarity in their flanking regions.

The characteristics of the SSR loci in *B. mori*, and generally in all Lepidoptera, have made identification and isolation of these markers difficult. They have also been a limitation for applications in genetic mapping. The low frequency and high homology (low percentage of polymorphic loci) of the microsatellites between contrasting lines of *B. mori*, possibly due to the size homoplasy, has not allowed the development of high-density linkage maps. This, together with the absence of mapping models adjusted to this organism, has hindered identification of genes and QTLs, limiting contributions mainly to preliminary mapping, for example of regions related to pigmentation patterns and development processes, as well as to weight of cocoon, cortex, pupa and filament.

These regions of repetitive DNA, however, have shown a high discriminating power between *B. mori* lines due to the high level of polymorphism, the finding of a percentage of single alleles higher than 20%, and the high levels of homozygosity in the materials analyzed, so that a reduced subset of 5–8 SSR loci have made it possible to generate DNA fingerprints, estimating the genetic diversity of domesticated and wild materials and determining the genetic relationships between closely related lines such as Dazao and P50. Although these markers additionally represent a potential tool for identifying *B. mori* cell lines, it is necessary to evaluate and select a subset of microsatellites with relatively low mutation rates that provide stability to the genetic profiles for 200 or more subcultures.

The data indicate that microsatellites will continue to be important for the study, management, conservation and use of silkworm germplasm. They have shown superior performance in these aspects compared to most molecular markers and are versatile – they can be analyzed, depending on resources available and expected reliability, with traditional polyacrylamide gels, with analysis of DNA fragments marked with fluorescence, or with the latest sequencing technologies. The latter, in addition to providing greater precision and automation in genotyping, would also facilitate identification of suitable SSR loci as molecular markers and allow simultaneous analysis with single nucleotide polymorphisms (SNPs), which would complement and strengthen the inferences and analyzes obtained when using them separately.

In this context, microsatellites would play an important role both in supporting the research carried out in *B. mori* germplasm banks in emerging countries wishing to promote sericulture in their territories, but that do not have the resources to access cutting-edge technologies and in advancing understanding of the complex genetic and molecular mechanisms underlying characteristics of productive and biological interest.

## Data availability

No data are associated with this article.
