# Supplementary material for: Role of microsatellites in genetic analysis of Bombyx mori silkworm: a review
Source: F1000Res. 2019 Aug 13;8:1424. [Version 1] doi: 10.12688/f1000research.20052.1 (PMC7043130; doi:10.12688/f1000research.20052.1)
Supplement: Peer review history

# Peer review: Reviewer response for version 1

**DOI:** 10.5256/f1000research.22017.r59560

**Published:** 2020-02-24

**Authors:** Maria-Lucia Carneiro Vieira

**Affiliations:** 1 Departamento de Genética, Escola Superior de Agricultura “Luiz de Queiroz”, Universidade de São Paulo, São Paulo, Brazil

I do believe that the review should be useful for readers, it considers the state of the art on the role of microsatellitesfor the genetic analysis of *Bombyx mori *silkworm. The most recent citation dates from 2018; therefore, I suggest authors to search for more recent articles on other Lepidoptera of agronomic importance. The population of these pests tend to have the same genetic structure as the ones of *Bombyx mori *silkworm? Please make the comparison.

I confirm that I have read this submission and believe that I have an appropriate level of expertise to confirm that it is of an acceptable scientific standard.

## References

[1] : Microsatellite markers: what they mean and why they are so useful. *Genet Mol Biol*.39(3) : 10.1590/1678-4685-GMB-2016-0027 312-28 10.1590/1678-4685-GMB-2016-0027 27561112PMC5004837

# Peer review: Reviewer response for version 1

**DOI:** 10.5256/f1000research.22017.r54605

**Published:** 2019-10-28

**Authors:** Nalavadi Chandrakanth

**Affiliations:** 1 Silkworm Breeding and Biotechnology, Central Sericultural Research and Training Institute, Berhampore, India

The authors have scripted the manuscript properly and covered the appropriate and relevant literature necessary for understanding the characteristics of microsatellite and the evolutionary events associated with it in *Bombyx mori* genome. Further this review has focused on the literature pertaining to the applications of the microsatellite as markers, in genetic analysis studies and in construction of the linkage maps in *B. mori*. Interestingly, the authors have also discussed about the lacuna of using microsatellite in marker-trait association studies and construction of high density linkage maps in *B. mori*. The authors have brought out an important point that linkage map construction in silkworm cannot be adjusted to the other models due to their gender based effects and absence of chromosomal cross-linking in the germinal line of the female silkworms. In this line, recently proposed statistical models to analyze QTLs were also reported and explained in this manuscript.

**Comments**

 In the last paragraph of Introduction, it was mentioned that this review will focus on the roles or strategies involving microsatellite markers for conservation of silkworm germplasm. But, in future scope, this part has to be included with effective strategies to conserve the silkworm germplasm using microsatellite markers. In addition, the authors can also report in the future scope about how these microsatellite markers has been employed in breeding programs to improve the genetic materials of different silkworms.  

 In some parts of the manuscript, the sentences are very lengthy which can be simplified.

I confirm that I have read this submission and believe that I have an appropriate level of expertise to confirm that it is of an acceptable scientific standard.
